# The Value of a BP Determination Method Using a Novel Non-Invasive BP Device against the Invasive Catheter Measurement

**DOI:** 10.1371/journal.pone.0100287

**Published:** 2014-06-23

**Authors:** Jinsong Xu, Yanqing Wu, Hai Su, Weitong Hu, Juxiang Li, Wenying Wang, Xin Liu, Xiaoshu Cheng

**Affiliations:** 1 Research Institute of Cardiovascular Diseases and Department of Cardiology, Second Affiliated Hospital of Nanchang University, Nanchang, Jiangxi, People' Republic of China; 2 Fuzhou Medical College of Nanchang University, Fuzhou, Jiangxi, People's Republic of China; Temple University, United States of America

## Abstract

**Objective:**

The aim of this study was to evaluate the accuracy of a new blood pressure (BP) measurement method (Pulse method).

**Methods:**

This study enrolled 45 patients for selective percutaneous coronary intervention (PCI) via right radial artery. A BP device using either oscillometric (Microlife 3AC1-1) or Pulse method(RG-BP11)was used. At the beginning of each PCI, intra-radial BP was measured before Microlife BP or Pulse BP measurement as its own reference, respectively. At the end of PCI, BP was measured again with the measurement order of Microlife BP and Pulse BP reversed. The differences between intra-radial and Microlife (BPi-M) or Pulse BP (BPi-P) on SBP, DBP and mean artery pressure (MAP) were calculated. Meanwhile, in 48 patients the intra-brachial BP and intra-radial artery BP were measured to calculate the brachial -radial BP difference (BPr-b).

**Results:**

The intra-radial SBP references used prior to both the Microlife and Pulse SBP that were similar (145.1±27.7 vs 145.8±24.2 mmHg), but the Microlife SBP was significantly lower than the Pulse SBP (127.7±20.5 vs 130.3±22.7 mmHg, P<0.05), thus the SBPi-M was higher than SBPi-P (18.1±11.8 vs 14.8±12.8 mmHg, P<0.05). As the mean SBPr-b was 12.4 mmHg, the Pulse SBP was closer to expected intra-brachial SBP by about 3.3 mmHg than was Microlife SBP to expected intra-brachial SBP. Meanwhile, Bland-Altman plots showed that the 95% limits of agreement for intra-radial SBP by Pulse SBP were narrower than those by Microlife SBP (12.0∼17.5 vs 15.5∼20.6 mmHg). However, the 95% limits of agreement for Pulse DBP and MAP were similar to those for Microlife DBP and MAP.

**Conclusion:**

Against the invasive BP measurement, the pulse method may provide more accurate SBP and comparable DBP and MAP as compared with the oscillometric method.

## Introduction

Mercury sphygmomanometers will be replaced by new blood pressure (BP) devices in the near future because of their pollution problems. At present, most electronic automatic BP devices use the oscillometric method to determine systolic and diastolic BP (SBP and DBP). Although electronic automatic BP devices are widely used in clinical practice, controversy about their accuracy still exists [1]–[4].

Recently, some new non-invasive BP devices have been developed [5]–[7]. Among them, a novel automatic BP device (Pulse BP device) was developed and commercialized in China. This device uses a new “Pulse method” to determine SBP and DBP. Although this new BP device was patented in China and other countries [8], [9], its accuracy against invasive catheter BP has not been fully determined. The aim of this study is to compare the accuracy between the Pulse method and the oscillometric method against invasive catheter BP measurement.

### Subjects and methods

The proposal and consent procedures of this study were approved by the Ethic Committee of the Second Affiliated Hospital of Nanchang University. For selective percutaneous coronary intervention (PCI) and BP measurement, all patients provided their written informed consent.

#### Subjects

From May to October of 2013, 45 patients undergoing PCI via the right radial artery were enrolled for this study. All of the patients enrolled were subjected to their first PCI via the right radial artery and had stable hemodynamics during the PCI.

We excluded any patients who experienced acute myocardial infarction, aortic coarctation, congenital heart disease, acute heart failure, hemiplegia, pulseless disease, previous trans-radial PCI or had arrhythmia at BP measurement. The information of the subjects was summarized in Table 1.

**Table 1:** Summary of the information of the 45 patients.

Age(y)	69.4±20.1
HR(bpm)	72.7±12.8
SBP(mmHg)	127.6±20.5
DBP(mmHg)	95.1±15.2
Diabetes (%)	15(33.3)
Male (%)	34(75.6)
CHD (%)	33(73.3)

#### BP measurement and devices

Two types of automatic BP device were used in this study. One is by oscillometric method (Microlife BP 3AC1-1), and the other is by Pulse method (RG-BP11, the Ruiguang medical equipment co., LTD Shenzhen, China).

**Figure 1:**
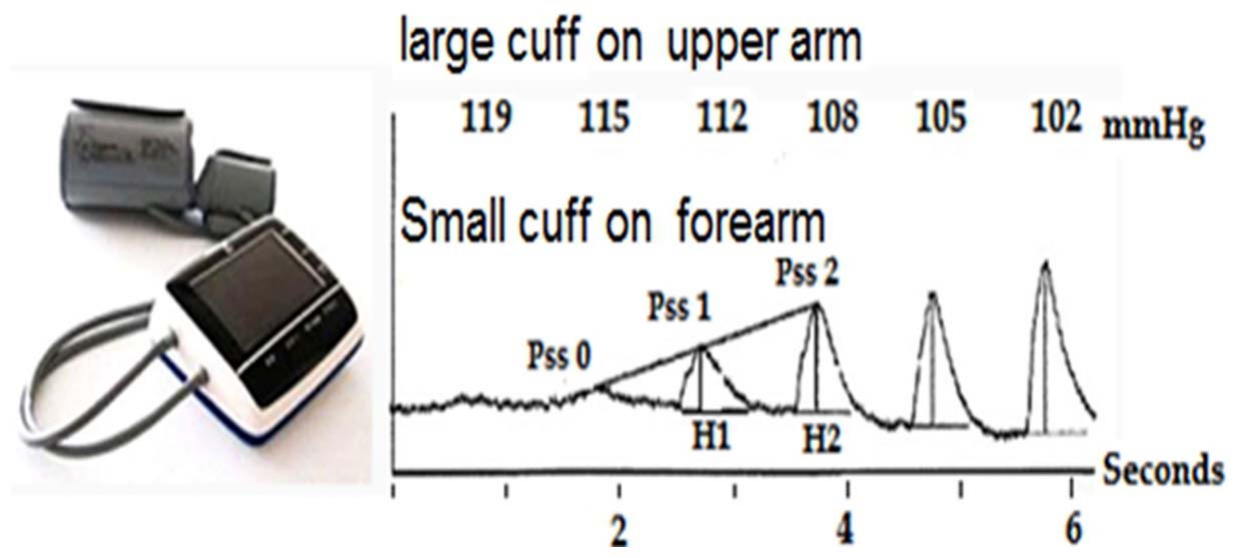
A Pulse BP device and the illustration for SBP determination. The numbers represent the pressure values (mmHg) in the large cuff. When the pressure decreases in the large cuff, the amplitudes of the first several pulse waves recorded from the small cuff gradually increases in a linear manner. Pss 0, Pss 1 and Pss 2 represent the first 3 pulse waves, respectively. H represents the vertical height of the pulse wave. The pressure in the large cuff at Pss0 is determined as SBP. Pss0 =  (H2 X Pssl -Hl X Pss2)/(H2— HI).

As a new device, the Pulse BP device has two features: First, it is equipped with two cuffs. The large cuff (14×27 cm) is placed on the middle of upper arm to compress brachial artery and to detect pressure, and the small cuff (7×23 cm) is placed on forearm near the fossa cubitalia to detect pulse wave of radial artery. The pressure curve in the large cuff and the pulse wave curve in the small cuff are simultaneously recorded. The second feature is the new determination method for SBP and DBP. When the pressure in the large cuff on upper arm decreases to the level that no longer blocks the brachial artery blood flow, pulse wave appears in the small forearm cuff. As the pressure decreases, the amplitudes of pulse waves in the small cuff gradually increase in a linear manner for the first several beats. The pressure in the large cuff that correlates with the cross point of the baseline line with the regression line in the small cuff is determined as SBP (Figure 1).

The determination of DBP is based on delay time, which is defined by the interval between the fluctuation signal in the large cuff and pulse wave in the small cuff. As the large cuff gradually deflates, the delay time gradually decreases. When the delay time becomes constant, the pressure in the large cuff is determined as DBP(Figure 1).

### The BP measurement protocol

The examination was conducted in cardiac catheterization room. BP was taken when the patients lay on an operation table with right arm on a support pad. The large cuff of the Pulse BP device was also used for the Microlife BP device with a T-branch pipe in order to eliminate the effect of cuff size on BP measurement.

At first, the two cuffs of the Pulse BP device were properly placed according to the manufacturer's instructions. After sheathing (5Fr or 6Fr), a Judkins catheter was inserted into the lower edge of the small cuff at the position of the upper-middle part of the radial artery. When intra-radial BP curves became stable, the mean amplitude of ten pulse waves was recorded as intra-radial BP, and Pulse BP was taken subsequently. After 2 minutes, intra-radial BP curve was recorded again, followed by the reading of Microlife BP. At the end of PCI, the above-mentioned BP measurement procedure was repeated when the catheter was withdrawn back to the lower edge of the small cuff, while the order of two non-invasive BP devices was reversed. Therefore, both Microlife BP and Pulse BP had its own intra-radial artery BP as its gold standard.

The non-invasive mean arterial pressure (MAP) was calculated with the formula: MAP =  (SBP+2×DBP)/3. The invasive MAP was read from the intra-artery pulse wave record (EP-Work Mate). The differences between intra-radial artery BP and Microlife BP (BPi-M) or Pulse BP (BPi-P) were calculated, respectively.

Meanwhile, in 48 patients (including the 20 patients received both Pulse and Microlife BP measurement) intra-brachial artery BP was recorded when the catheter was withdrawn to the middle of brachial artery in order to evaluate the difference between the intra-radial BP and the intra-brachial BP (BPr-b).

### Statistical analysis

Data was entered in Excel 2003 and analyzed with SPSS10.0. Continuous variables were expressed as mean ± SD. The t-test, paired sample t-test and the analysis of variance (ANOVA) and the omnibus test were used for the statistical analysis. Linear regression analysis was performed to examine the correlation between BPs from invasive and noninvasive methods.

The inter-measurement agreement was evaluated by Bland-Altman plot method [10]. With this method, inter-measurement differences for SBP, DBP and MAP were plotted against their relative intra-radial artery parameters, respectively. The 95% limits of agreement (LoA) were determined (95% LoA  =  mean inter-measurement difference ±1.96 standard deviation). A p-value of less than 0.05 was considered statistically significant.

## Results

As 4 patients had no complete paired Pulse and Microlife BP data, only 85 pairs of intra-radial and Microlife BPs and 85 pairs of intra-radial and Pulse BPs were finally used for analysis. The range (93–206 vs 88–226 mmHg) and the mean value of intra-radial SBP taken before Microlife BP were very similar to those taken before Pulse BP. However, the Microlife SBP was significantly lower than the Pulse SBP, so the SBPi-P was significantly lower than the SBPi-M. Significantly positive correlation was seen between intra-radial SBP and Pulse SBP (r = 0.89) or Microlife SBP (r = 0.87).

The range (49–102 vs 49–108 mmHg) and the mean value of intra-radial DBP taken before Microlife BP were also very similar to those taken before Pulse BP. Both Pulse DBP and Microlife DBP were significantly higher than their reference intra-radial DBPs, while the DBPi-P was similar to the DBPi-M. Meanwhile, the coefficient of the intra-radial DBP with Microlife DBP was similar to that with Pulse DBP (0.81 vs 0.79).

The reference intra-radial MAP for Pulse MAP was similar to that for Microlife MAP, although the MAPi-P was slightly lower than the MAPi-M. Nevertheless, the coefficients between the intra-radial MAP and Microlife MAP or Pulse MAP were nearly equal (Table 2).

**Table 2:** The comparison between intra-radial and Microlife or Pulse BP.

	Microlife			Pulse		
	SBP	DBP	MAP	SBP	DBP	MAP
Intra-radial	145.8±24.2	69.9±10.2	95.2±12.8	145.1±27.7	70.5±11.6	95.4±14.9
Noninvasive	127.7±20.7*	74.6±10.9*	92.3±12.7*	130.3±22.7*	75.8±12.1*	94.0±13.9
BPi-n	18.1±11.8	−4.7±6.5	2.9±6.6	14.8±12.8^$^	−5.3±7.7	1.4±7.1
R	0.87^#^	0.81^#^	0.87^#^	0.89^#^	0.79^#^	0.88^#^

BPi-n: the difference between the intra-radial artery and noninvasive BP;

*: compared with intra-radial artery BP, P<0.05; &: compared with Microlife SBP, P<0.05;^#^: the coefficient, P<0.001;

(n = 85,M±SD,mmHg).

In the 48 patients with both intra-radial and intra-brachial BP measurements in right arm, the intra-radial SBP and MAP were significantly higher than the intra-brachial SBP and MAP, while their intra-radial DBP was similar (Table 3).

**Table 3:** The comparison between intra-radial and intra-brachial artery BPs.

	intra-radial	intra-brachial	BPr-b
SBP	146.0±25.4	133.5±22.8*	12.4±8.2
DBP	74.4±14.4	73.2±13.5	1.2±6.6
MAP	98.3±15.7	93.3±14.1*	5.0 ±6.0

BPr-b: the difference between intra-radial and intra-brachial artery BP.

*: compared with intra-radial, P<0.05.

(n = 48, mmHg).

**Figure 2:**
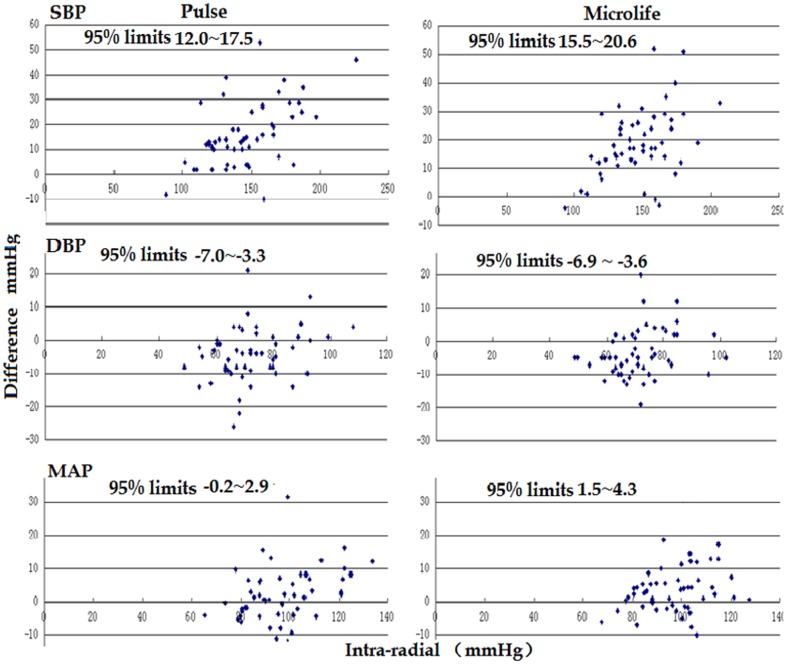
The Bland-Altman plots for the inter-measurement differences between intro-radial artery BP and Pulse BP or Microlife BP. Difference  =  intra-radial BP- non-invasive BP.

Figure 2 shows the Bland-Altman plots for the intra-radial BP and the non-invasive BP. The 95% limits of agreement for Pulse SBP (12.0∼17.5 vs 15.5∼20.6 mmHg) and Pulse MAP (−0.2∼2.9 vs 1.5∼4.3 mmHg) were narrower than those for Microlife SBP and MAP.

However, the 95% limits of agreement for Pulse DBP (−7.0∼−3.3 vs −6.9∼−3.6 mmHg) were similar to those for Microlife DBP (Figure 2).

In the Bland-Altman plots, a positive linear correlation is observed between SBP and SBP difference for either Pulse (r = 0.58, P<0.01) or Microlife (r = 0.52, P<0.01) methods. Meanwhile, the coefficient is 0.27 for Pulse (P = 0.012) and 0.21 for Microlife (P = 0.049) between DBP and DBP difference, and the coefficient is 0.33 for Pulse (P = 0.002) and 0.24 for Microlife (P = 0.024) between MAP and MAP difference (Figure 2).

## Discussion

The Pulse BP device used in this study has passed the validation process against sphygmomanometers according to the European Society of Hypertension International Protocol (by Doctor Wang W of Fuwai hospital, Beijing, China). This study aimed to evaluate the accuracy of Pulse BP method against intra-artery BP measurement.

The present study showed that at similar reference intra-radial SBP, the mean difference between intra-radial SBP and Pulse SBP was 14.8 mmHg, lower than the difference of 18.0 mmHg between intra-radial SBP and Microlife SBP. Meanwhile, this study confirmed SBP amplification phenomenon between intra-radial and intra-brachial artery [11], [12], and this value was 12.4 mmHg in the 48 patients examined. When the SBP amplification phenomenon was taken in to consideration, the Pulse SBP was about 3 mmHg closer to the expected intra-brachial SBP as compared with Microlife SBP. Furthermore, the 95% limits of agreement of differences between intra-radial SBP and Pulse SBP were narrower than those between intra-radial SBP and Microlife SBP, and the coefficient of intra-artery SBP with Pulse SBP was slightly higher than that with Microlife SBP (0.89 vs 0.87). These results indicate that the Pulse method may provide more accurate brachial artery SBP as compared with the oscillometric method. For the oscillometric method, a state-of-the-art electronic measurement method, SBP is estimated by the average pressure and the empirical coefficient. Therefore, the reported BP may have relatively large individual differences, especially in some extreme clinical situations such as severe hypertension or hypotension.

This study showed that the two methods had comparable accuracy on MAP. After the correction for the MAP amplification phenomenon of 5.0 mm Hg, the Microlife MAP and the Pulse MAP were 2.1 and 3.6 mmHg higher than the expected intra-brachial MAP, respectively. Although the Microlife MAP was closer to the expected intra-brachial MAP by 1.5 mmHg, its 95% limits of agreement with intra-radial MAP were wider than those with Pulse MAP (1.5∼4.3 vs −0.2∼2.9 mmHg). Furthermore, their coefficients for intra-radial MAP were nearly equal; therefore, we suggest that these two methods have similar accuracy on MAP.

Although the Pulse BP device also uses a new delay time method, our study did not show that this method had advantage on DBP determination as compared with the oscillometric method, as similar coefficients and 95% limits of agreement were observed between intra-radial and Pulse or Microlife DBPs. Meanwhile, this study showed no amplification phenomenon on DBP between brachial artery and radial artery.

In the Bland-Altman plots a positive linear correlation is observed between intra-radial SBP and SBP difference (intra-radial SBP- non-invasive SBP), between DBP and DBP difference, and between MAP and MAP difference for both methods. These results suggest that when the intra-radial BP is higher, the noninvasive brachial BP may be underestimated by both BP measurement methods, inducing higher intra-radial BP- non-invasive BP difference, especially for SBP. Meanwhile, the stronger correlation for Pulse method may indicate that the Pulse BP is more closely correlated with the intra-radial BP than with Microlife BP, especially for SBP.

### Limitation

For ethical reasons, only some of the 45 patients who received both Pulse and Microlife BP measurement had intra-brachial artery BP values. The BP difference between intra-radial and intra-brachial artery used as the reference was partly obtained from other 48 patients, so the use of their data as the gold standard may result in some bias. However, the intra-radial SBP and DBP were very similar in the 2 groups of patients studied, suggesting this system error may not influence our conclusion.

## Conclusion

Against the invasive BP measurement, the pulse method may provide more accurate SBP and comparable DBP and MAP as compared with the oscillometric method.
